# Supplementary material for: Do pregnant African women exercise? A meta-analysis
Source: PLoS One. 2023 Sep 8;18(9):e0289421. doi: 10.1371/journal.pone.0289421 (PMC10490998; doi:10.1371/journal.pone.0289421)
Supplement: S2 Table — (DOCX) [file pone.0289421.s002.docx]

S2 table: NOS quality assessment of included studies on do African women exercise?

| Studies | **Quality assessment criteria** | | | |
| --- | --- | --- | --- | --- |
|  | **Selection** | **Comparability** | **Outcome** | **Overall quality** |
| A.Nkhata et.al ([**27**](#_ENREF_27)) | **** | * | ** | 7 |
| Addis et.al ([**28**](#_ENREF_28)) | **** | ** | ** | 8 |
| Bayisa et.al ([**29**](#_ENREF_29)) | **** | * | ** | 7 |
| Beyene et.al ([**22**](#_ENREF_22)) | **** | ** | ** | 8 |
| DRC.C et.al ([**30**](#_ENREF_30)) | **** | ** | ** | 9 |
| J.I.B et.al ([**7**](#_ENREF_7)) | **** | ** | ** | 8 |
| Janakiraman et.al ([**31**](#_ENREF_31)) | **** | ** | ** | 8 |
| Mbada et.al ([**32**](#_ENREF_32)) | **** | ** | ** | 8 |
| Mervat et.al ([**33**](#_ENREF_33)) | **** | * | ** | 7 |
| Ngayimbesha et.al ([**5**](#_ENREF_5)) | **** | * | ** | 7 |
| Sitot et.al ([**9**](#_ENREF_9)) | *** | ** | ** | 7 |

NOS: Newcastle Ottawa Scale

** Two points, *** Three points; and **** four point
