# Supplementary material for: Do pregnant African women exercise? A meta-analysis
Source: PLoS One. 2023 Sep 8;18(9):e0289421. doi: 10.1371/journal.pone.0289421 (PMC10490998; doi:10.1371/journal.pone.0289421)
Supplement: Attachment — Submitted filename: Response to Reviewers.docx [file pone.0289421.s005.docx]

Dear reviewer, we are excited to incorporate your insightful criticism to improve our article. As a result, we changed the manuscript; the changes are indicated in the revised version by track changes. Below are point by point responses to the reviewer concerns.

Reviewer #1: I would like to appreciate the authors for their effort to come up with this interesting subject which has paramount importance in pregnant women health. As life style gradually becoming sedentary both in developed and developing countries, assessing prevalence of exercise particularly among pregnant women is topic need to studied. I have reviewed the document thoroughly and seen the document was well done. However, some of basic things in meta-analysis study need to be Clearfield. My comments and questions that author should address are listed herewith
Methods
Study design and setting
Prospero's registration number should be indicated.

**Response:** It’s mentioned in the current version of the manuscript.

Eligibility criteria
If the current meta-analysis included all studies conducted in any African country, how have you accessed those studies published in a language other than English?

**Response**: Not at all; studies published in a language other than English were excluded from this study.
It is not easy to incorporate unpublished master’s thesis and PhD dissertations from repositories in whole African countries. So, what strategies were used to get those studies should be clarified under search strategy.

**Response**: Search strategy of this study was revised in the current version.
Some of the information under inclusion criteria is about study selection. So, it is better to give it a separate title and describe it with a figure.

**Response**: Separate sub-section for study selection was given under the methods and materials section, and it’s already described in figure 1.
Quality assessment
What are the criteria considered to assess the quality of the articles included in the study? It was indicated that you have used the Newcastle-Ottawa scale (NOS). But what strategy have you used to assess the scale? Is it done by one of the authors or an independent assessment? What was the score of the assessment? Is there any article of poor quality? If yes, what has been done?

**Response**: All articles were independently verified by the two authors (YFG and SJM). Where disagreements have occurred, the articles have been reviewed by one of the authors (TMB) and used as final mediation and admissibility decision. The NOS was included as a supplementary table 2 (S_table2) in the current submission. As it was described on the exclusion criteria studies with poor qualities like articles that did not have proven methods, articles that do not contain comprehensive information of importance for analysis and case reports and articles with inconsistent measures of outcome variables were excluded from the final analysis of this study.
Data analysis
The author declared that there was significant heterogeneity observed across studies and used a random effect model for it. It is obviously an expected event, as you are collecting information from the whole continent of Africa. Hence, analysis to understand the source of heterogeneity will be helpful. It guides the author to do subgroup analysis. So, how do you detect possible sources of heterogeneity, and have you done the subgroup analysis? Is the source of heterogeneity is the country?

**Response**: Yes, in this investigation, national differences were discovered to be one of the sources of heterogeneity among the studies considered.
I was wondering if I could see the list and definition of your study variables in the methods section, but I could not. How do you define your outcome variable "exercises during the antenatal period"? How the primary studies measured exercise during pregnancy. As we know, most pregnant women in Africa remain active even in late pregnancy due to their lifestyle. So, how do you differentiate this routine lifestyle physical activity from what we call two or three days of exercise in a week?

**Response**: The "operational definition" was included in the methods and materials section of the most recent version of the manuscript. There can be no denying that pregnant women in rural Africa engage in strenuous physical activity such as carrying, fetching, and other tasks. Contrarily, compared to rural women, pregnant African women live opposite lifestyles in urban areas. Furthermore, it may be difficult to argue that pregnant African women living in rural areas do not require pre-planned physical activity. Due to these and the reviewer's concerns, we already recommended conducting a comparative meta-analysis.
Conclusion and recommendation
The objective/result of your study was the pooled prevalence of physical exercise among pregnant women. You have not assessed any factor associated with it, however.
You are recommending
To change policies and maternal health guidelines, and health promotion activities in Africa. How do you know these are the factors contributing to low physical activity among pregnant women? Your recommendation should be in line with your work.

**Response**: The recommendation was revised in the current submission.
lastly, grammar and punctuation of the manuscript should be rechecked

**Response**: Grammar and punctuation of the manuscript were thoroughly revised in the current version.

Thank you!
